# miR-96 attenuates status epilepticus-induced brain injury by directly targeting Atg7 and Atg16L1

**DOI:** 10.1038/s41598-017-10619-0

**Published:** 2017-08-31

**Authors:** Jing Gan, Qianyun Cai, Yi Qu, Fengyan Zhao, Chaomin Wan, Rong Luo, Dezhi Mu

**Affiliations:** 1 Department of Pediatrics, West China Second University Hospital, Sichuan University, Chengdu, 610041 China; 2 Key Laboratory of Obstetric & Gynecologic and Pediatric Diseases and Birth Defects of Ministry of Education, Sichuan University, Chengdu, 610041 China; 3 Department of Pediatrics, University of California, San Francisco, CA94143 USA

## Abstract

Status epilepticus (SE) can cause brain damage and lead to neural dysfunction. Developing novel targets for SE therapy and diagnosis is important and necessary. Previously, we found several differentially expressed microRNAs (miRNAs) in the developing hippocampus following SE, including the autophagy-related miR-96. In the present study, we employed immunofluorescence staining and Western blot analysis to assess the expression of autophagy-related 7 (Atg7) and Atg16L1 and the status of autophagosome formation in the hippocampus of immature rats with SE. Additional *in vivo* intervention was also performed to investigate the potential therapeutic function of miR-96 in developing rats with SE. We found that Atg7 and Atg16L1 were up-regulated in the neurons after SE, together with an increase in autophagosome formation. Meanwhile, overexpression of miR-96 significantly prevented brain damage in SE rats by inhibiting Atg7 and Atg16L1 expression and autophagosome formation in the hippocampus. Furthermore, Rapamycin negated miR-96 mediated brain injury attenuation through inducing autophagosome formation. Our study indicates that miR-96 might be a potential target for therapy of pediatric SE.

## Introduction

Epilepsy is a chronic neurological disorder characterized by recurrent unprovoked seizures, often resulting from abnormal and highly synchronous neuronal discharges within the brain^1^. Increasing evidence has shown that epileptogenesis results in eventual neuronal death or dysfunction, ion channel dysfunction, mossy fibre sprouting, gliosis, neurogenesis, and inflammation, among other effects^2, 3^. The prolonged, non-terminating seizure known as status epilepticus (SE) is a neurological disorder that has the potential to cause irreversible brain damage and long-term cognitive deficits^4^. Clinical studies have reported that SE is the most severe and most deadly form of epilepsy^5–7^. About 5% of adults and 10–25% of children with epilepsy are predicted to have SE at least once in their lifetime^5^. The mortality rates in SE are high: 24–26% among adults and 3–6% among children, with an mean mortality rate of about 20%^7^. Hence, there is an increasing need for novel biomarkers and therapeutic targets for SE.

Autophagy refers to a process in which cellular components and pathogens are delivered to the lysosome for degradation, for the maintenance of cellular homeostasis^8^, having been shown to participate in both physiological and pathological processes^9^. Under physiological stress, the clearance of ubiquitylated, misfolded proteins or damaged organelles is mediated by autophagy. Additionally, impaired autophagy is also implicated in pathological processes, such as cancer^10, 11^, heart disease^12^, autoimmune disease^13^, and neurological disorders^14–16^, including epilepsy^17^. The LC3-II/LC3-I ratio and the expression of Beclin-1 have been shown to increase by recurrent neonatal seizures, and inhibiting these effects with cathepsin B inhibitor (CBI) plays a role in the prevention of seizure-induced neurobehavioral deficits via autophagy pathways^18^. Therefore, autophagy has been proposed as a therapeutic target for the treatment of neurological diseases.

MicroRNAs (miRNAs), short noncoding RNAs that are 18 to 22 nucleotides in length, have been shown to regulate gene expression at posttranscriptional levels by base pairing with targeted mRNAs^19^. Owing to the potential of each miRNA to suppress the expression of hundreds of genes^20^, miRNA-mRNA interactions form a complex gene regulatory network. Disease-associated miRNAs represent a new class of diagnostic markers or therapeutic targets^21^. Previous studies have indicated that the central nervous system (CNS) expresses the richest diversity of miRNAs among all human tissues^22^, and that miRNAs could be used as diagnosis and treatment targets for neural damage. Our previous study has also revealed close associations between miRNAs, autophagy and epilepsy^17^. By miRNA array and differential analysis, 29 up-regulated and 20 down-regulated miRNAs were identified in developing rat hippocampi^23^. Targets of these deregulated miRNAs were analysed using the miRWalk database, showing that the identified autophagy signalling pathway was involved in the molecular mechanisms underlying epileptogenesis^23^. miR-96 is one of the miRNAs showing significantly differential expression and correlated with the autophagy signalling pathway^23^. In the present study, we further investigated the potential autophagy-related miR-96, which we propose can be used as a diagnostic and therapeutic target for SE in immature rats. Our study also suggests that miR-96 acquires its protective role during the development of SE, thereby supporting its role as a novel therapeutic target for SE.

## Materials and Methods

### Animal model of SE

All of the animal research was approved by the Sichuan University Committee on Animal Research. Female Sprague-Dawley rats with mixed-sex litters were acquired from the animal center of Sichuan University (Chengdu, China). The mothers were provided food and water and were housed in a temperature- and light-controlled facility until the pups were 25 days old. All experiments were performed in accordance with relevant guidelines and regulations of the Sichuan University Committee on Animal Research. Lithium chloride (125 mg/kg, Sigma) was intraperitoneally (i.p.) administered 18–20 hours prior to pilocarpine injection. Pilocarpine was then i.p. administered (40 mg/kg, Sigma). The severity of convulsions was evaluated by Racine’s scale, and only those animals that were defined as showing continuous behavioral seizure activity lasting at least 30 min with a score of 4–5 were used in this study. All of the SE rats were then intraperitoneally injected with diazepam (10 mg/kg) to terminate the seizure attacks. The control rats received an injection of the same amount of normal saline as a replacement for pilocarpine. For the rat miRNA array, 4 randomly selected post-SE rats were included in the experimental group (n = 4), and 4 randomly selected normal rats were included in the control group (n = 4). The animals were euthanized 24 h after SE onset. The hippocampus was quickly removed from the brain after decapitation and then preserved in RNAlater (−20 °C, 2 mL, Qiagen, Germany) for microarray analysis.

miR-96 mimics and negative control (NC) were purchased from Ribobio (Guangzhou, China) and 10 nmol miR-96 mimics or NC in 5 μl PBS was injected into the right cerebral hemisphere of SE insult rats using a 30-gauge needle with a 5-μl Hamilton syringe injections. Rapamycin was purchased from Selleckchem and injected into the right cerebral hemisphere of SE insult rats (3-day pretreatment preceding SE, 3 mg/kg, once a day) as previous study indicated^24^.

### Electron microscopy

To determine the autophagosome in hippocampus, the brains were perfused and fixed in 4% paraformaldehyde and 1% glutaraldehyde in 0.1 mol/ for 48 h L sodium cacodylate buffer (pH 7.2) overnight at room temperature. Following fixation, thin sections were cut, mounted onto copper grids, contrasted with Uranyl acetate and Reynold’s lead citrate, and examined as previous study indicated^25^. Twelve random area has been selected for autophagosome detection. And the number of autophagosome was analyzed.

### Immunofluorescence staining

To determine the expression and distribution of LC3, Atg7 and Atg16L1 in hippocampus, the brains were perfused and fixed in 4% paraformaldehyde for 48 h and then embedded by paraffin and sectioned 4-mm thick for double immunofluorescence. The primary antibody against LC3, Atg7 and Atg16L1 and mouse monoclonal antibodies against NeuN (Millipore, CA; 1:150) or GFAP (Millipore, CA, 1:80) was performed and followed incubated with a secondary antibody, either FITC- or TRITC-conjugated anti-rabbit or anti-mouse IgG (Santa Cruz Biothecology, USA). The nucleus was stained with 4′,6-diamidino-2-phenylindole (DAPI, Beyotime, Beijing, China). Olympus B × 600 microscope and Spot Fiex camera were performed to evaluate all specimens.

### Western blotting

At different time point after SE, the hippocampus from the right hemisphere were perfused and collected. Hippocampus from different groups were lysised with the RIPA lysis buffer (Merck Millipore, MA, USA) containing a protease inhibitor cocktail (Merck Millipore, MA, USA). The protein concentrations were measured with the BCA protein assay kit (Beyotime, Beijing, China). After adding the loading buffer, 20 μg total protein per samples of each group were loaded for western blotting. After blocking for overnight at 4 °C, the membranes were immunoblotted with various antibodies: Atg7, Atg16L1, Beclin-1 and LC3. β-actin was used as the internal loading control. ECL advance western blotting detection kit (Merck Millipore, MA, USA) was performed to detect the bound antibodies and the Image-Pro Plus (version 6.0; Media Cybernetics, Inc., Rockville, MD, USA) was used to quantify the densities of the protein signals. The relative expression of Atg7, Atg16L1, Beclin-1 and LC3 was analyzed.

### RNA isolation and quantification

RNA was isolated from the hippocampus with the miRNA Isolation Kit (Qiagen, Germany), in accordance with the manufacturer’s instructions. The purity and quantity of RNA were measured by NanoDrop (ND-1000 spectrophotometer, Thermo Scientific, Wilmington, DE, USA). The samples were used immediately or stored at −80 °C.

### qRT-PCR

Reverse transcription was performed on the isolated total RNA using a Reverse Transcription kit (Takara Bio, Inc., Otsu, Japan), and PCR was performed using a Real Time PCR kit (Takara Bio, Inc.). Reverse transcription was performed at 65 °C for 5 min, 30 °C for 10 min, 42 °C for 10–30 min and 92 °C for 3 min. The PCR conditions were as follows: denaturation at 94 °C for 2 min; amplification for 30 cycles of denaturation at 94 °C for 0.5 min, annealing at 60 °C for 0.5 min, and extension at 72 °C for 1 min; followed by a terminal elongation step at 72 °C for 10 min. The procedure was performed on a Bio‑Rad CFX96 thermal cycler (Bio-Rad Laboratories, Inc.). U6 was amplified as an internal control, the Ct value of each PCR product was calculated, and the fold changes were analyzed as previous study^26^. The r-miR-96–5p and r-U6 primers were supplied by RiboBio Technology (Guangzhou, China); the sequences were not supplied due to the rules of the company.

### Target prediction

Target mRNAs were predicted by the miRWalk database (http://www.ma.uni-heidelberg.de/apps/zmf/mirwalk/) and by other programs (miRanda, Sanger miRDB, RNAhybrid and Targetscan) on the most used prediction Web site (http://www.umm.uni-heidelberg.de/apps/zmf/mirwalk/predictedmirnagene.html). This module hosts all experimentally verified miRNAs information associated with the genes and pathways, as well as information about proteins known to be involved in miRNA processing. The list of targeted mRNAs, in the form of official gene symbols, was extracted from the miRWalk prediction results for KEGG analysis^27–29^.

### HE staining and nissl staining

The paraffin embedded 4-mm thick brain sections from different group were used for H&E staining and nissl staining. The hematoxylin, eosin and the nissl staining kit were supplied by Beyotime (Beijing, China). The sections were deparaffinized in xylene, treated with a graded series of alcohol [100%, 95%, and 85% and 75%, ethanol/double-distilled H_2_O (v/v)], and rehydrated in PBS (Supplied by Zsbio, Beijing, Ching). The HE and nissl staining were performed following the instructions supplied by the manufacturer. Olympus B × 600 microscope and Spot Fiex camera were performed to evaluate all specimens.

### Statistical analysis

Two-way ANOVA was performed for the miRNA profiles using GeneSpring software. Statistical analysis was performed by the Student’s t-tests for comparing two groups and by analysis of variance for multiple group comparisons. Statistical analyses and the Pearson correlation analysis were performed using SPSS software, version 19.0 (IBM SPSS, Armonk, NY, USA). Values are expressed as the mean ± standard error of the mean. P < 0.05 was considered to indicate a statistically significant difference.

## Results

### Autophagy increased in the hippocampus of rats with SE

**Figure 1:**
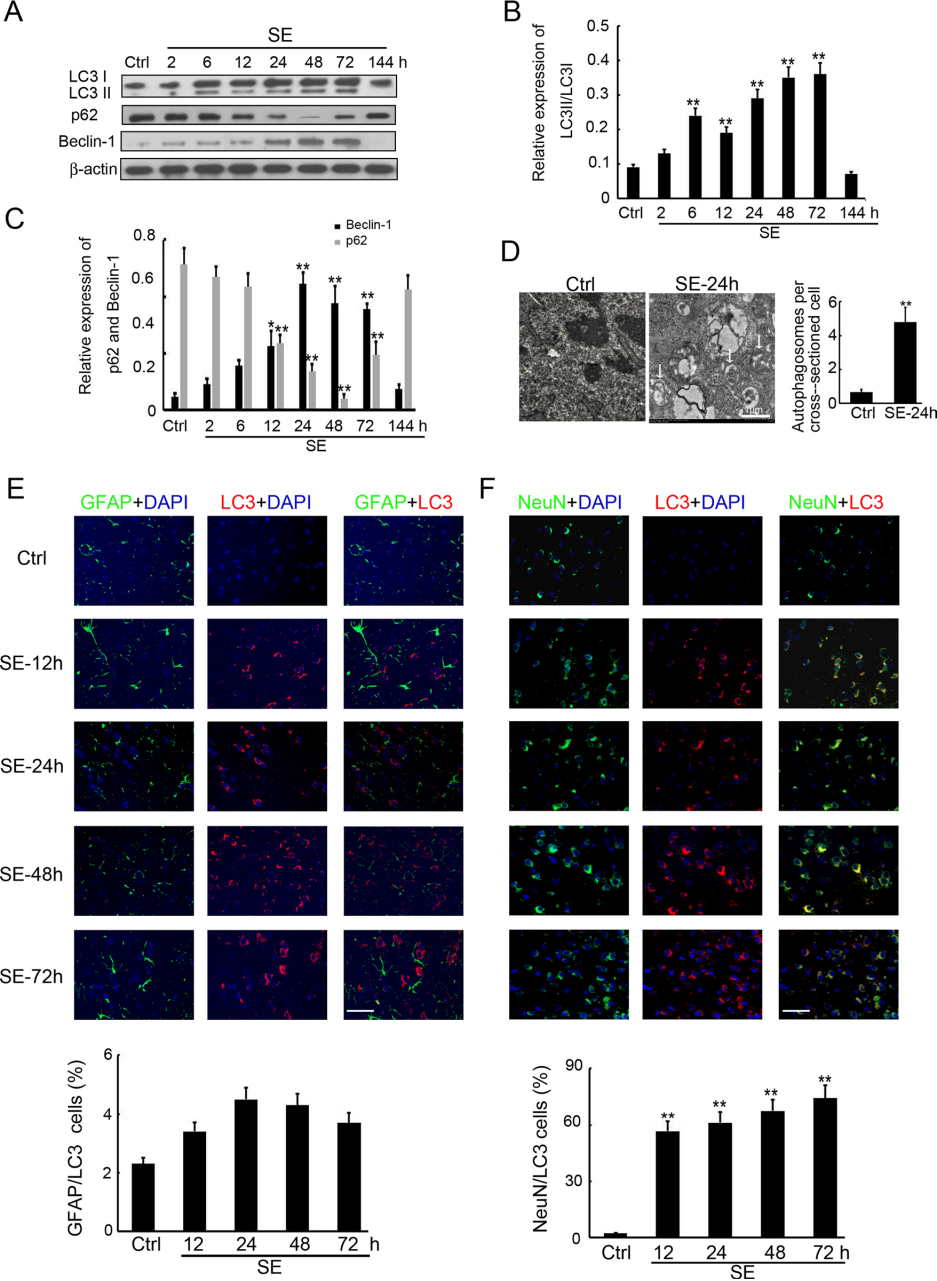
Autophagy was increased in the hippocampus of rats with SE. (**A**) Western blotting analysis of LC3, p62 and Beclin-1 expression in the hippocampus of SE rats. Equal levels of protein samples (20 μg) were loaded, and β-actin served as a loading control. (**B**) The relative optical density (LC3II/LC3I) were analyzed and represented as the mean ± SD (n = 3; **p < 0.01, compared with the ctrl group). (**C**) The relative optical density (p62 and Beclin-1) were analyzed and represented as the mean ± SD (n = 3; **p < 0.01, compared with the ctrl group). (**D**) Autophagosome in the hippocampus of SE rats and control rats were detected by electron microscopy. The number of autophagosome was analyzed. (n = 12; **p < 0.01, compared with the ctrl group). (**E**) The cell type specificity of LC3 was performed with double fluorescent immunolabeling (TRITC-labeled, red), accompanied with the astrocyte-specific marker GFAP (FITC-labeled, green). The nucleus was stained by DAPI (blue). Scale bar = 50 μm. The percent of GFAP and LC3 positive cells was analyzed (n = 5; ns, no significant difference compared with the ctrl group). (**F**) The cell type specificity of LC3 was performed with double fluorescent immunolabeling (TRITC-labeled, red), accompanied with the neuron-specific marker NeuN (FITC-labeled, green). The nucleus was stained by DAPI (blue). Scale bar = 50 μm. The percent of NeuN and LC3 positive cells was analyzed (n = 5; **p < 0.01, compared with the ctrl group).

We quantified the expression of LC3, p62 and Beclin-1, the cellular markers of autophagosomes^30^, in the hippocampus of rats with SE, by Western blot analysis. Total protein was isolated from the hippocampus of SE rats at 2, 6, 12, 24, 48, 72 and 144 h post insult. We found that the expression of LC3II/LC3I was dramatically up-regulated from 2 to 72 h post insult, peaking at 48 h and remaining elevated at 72 h, before reducing back to the basal level at 144 h post insult (Fig. 1A,B). Similarly, as shown in Fig. 1(A,C), Beclin-1 expression was also increased from 12 to 72 h after SE, peaking at 48 h and lowering back to the basal level at 144 h post insult. Meanwhile, p62 expression was decreased from 17 to 72 h after SE (Fig. 1A,C). Furthermore, autophagosome in the hippocampus of SE rats were detected by electron microscopy (Fig. 1D), but no autophagosome was found in the control group. To better investigate the expression and distribution of LC3 in the hippocampus after SE insult, double immunofluorescence staining was performed to detect this protein, together with the astrocyte-specific marker GFAP or the neuronal-specific marker NeuN as controls. The results indicated only a small amount of LC3 was found in astrocytes (Fig. 1E), whereas LC3-immunoreactive cells mainly colocalized with neurons (Fig. 1F). The above results showed a dramatic increase of LC3 expression, indicating the presence of autophagosomes in neurons after SE.

### Expression and distribution of Atg7 after SE

**Figure 2:**
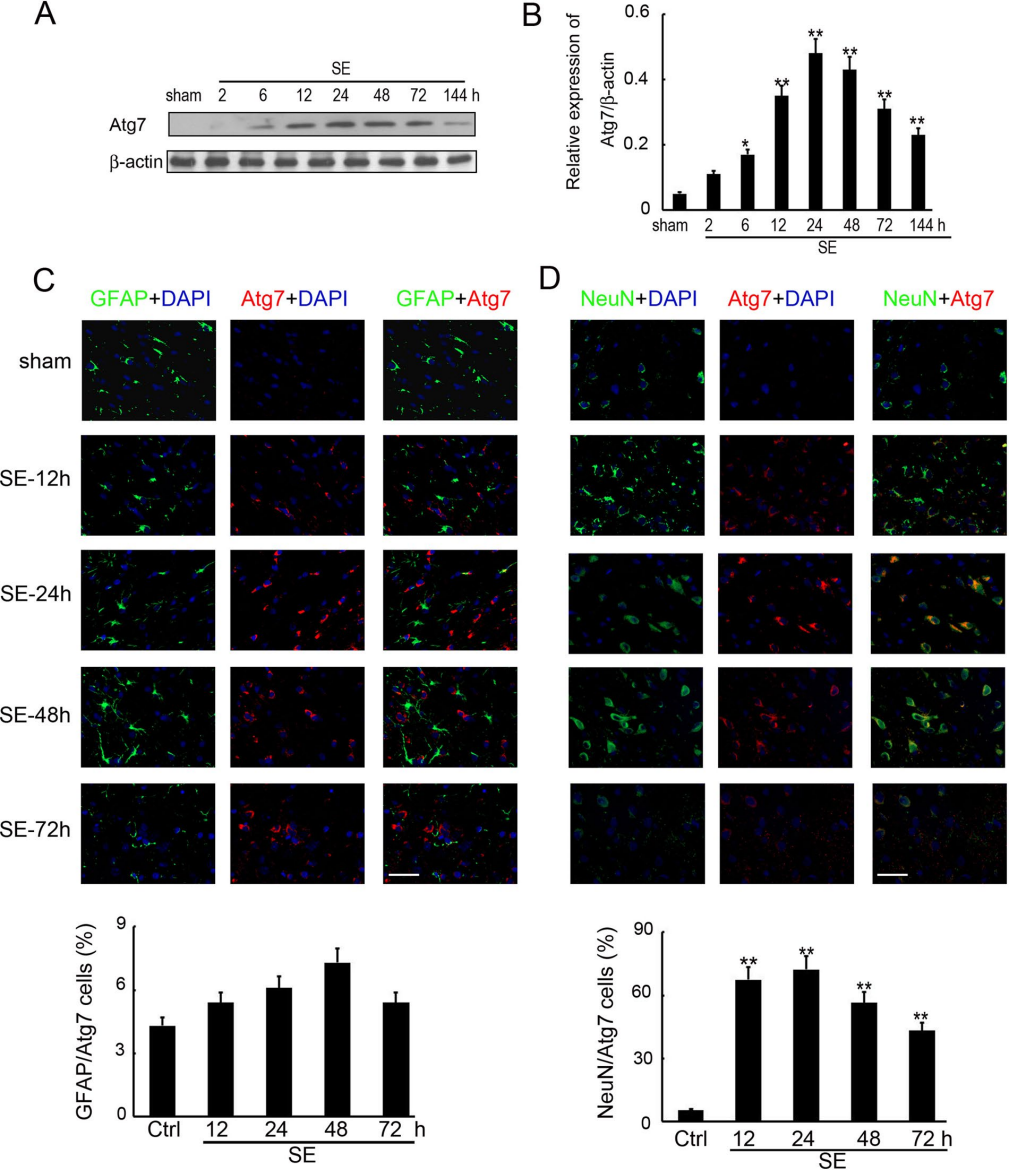
Expression and distribution of Atg7 after SE. (**A**) Western blotting analysis of Atg7 expression in the hippocampus of SE rats. Equal levels of protein samples (20 μg) were loaded, and β-actin served as a loading control. The relative optical density (Atg7/β-actin) were analyzed and represented as the mean ± SD (n = 3; **p < 0.01, compared with the ctrl group). (**B**) The cell type specificity of Atg7 was performed with double fluorescent immunolabeling (TRITC-labeled, red), accompanied with the astrocyte-specific marker GFAP (FITC-labeled, green). The nucleus was stained by DAPI (blue). Scale bar = 50 μm. The percent of GFAP and Atg7 positive cells was analyzed (n = 5; ns, no significant difference compared with the ctrl group). (**C**) The cell type specificity of Atg7 was performed with double fluorescent immunolabeling (TRITC-labeled, red), accompanied with the GFAP (FITC-labeled, green). The nucleus was stained by DAPI (blue). Scale bar = 50 μm. The percent of GFAP and Atg7 positive cells was analyzed (n = 5; **p < 0.01, compared with the ctrl group). (**D**) The cell type specificity of Atg7 was performed with double fluorescent immunolabeling (TRITC-labeled, red), accompanied with the neuron-specific marker NeuN (FITC-labeled, green). The nucleus was stained by DAPI (blue). Scale bar = 50 μm. The percent of NeuN and Atg7 positive cells was analyzed (n = 5; **p < 0.01, compared with the ctrl group).

We next investigated the expression of Atg7, which has a pivotal role in the formation of autophagosomes^31^, in the hippocampus of rats with SE, by Western blot analysis at 2, 6, 12, 24, 48, 72 and 144 h post insult. The results indicated a significant up-regulation of Atg7 from 6 to 144 h post insult, reaching the highest level at 24 h and remaining elevated at 48 h (Fig. 2A). To further determine the expression and distribution of Atg7 in the hippocampus after SE insult, double immunofluorescence staining was performed to detect Atg7, as well as the astrocyte-specific marker GFAP or the neuronal-specific marker NeuN. The immunofluorescence staining also indicated an increase of Atg7 positive cells in the hippocampus from 12 to 72 h post SE, further supporting the findings of Western blot analysis (Fig. 2C,D). While Atg7-immunoreactive cells mainly colocalized with neurons (Fig. 2D), only a small amount was found in astrocytes (Fig. 2C).

### Expression and distribution of Atg16L1 after SE

**Figure 3:**
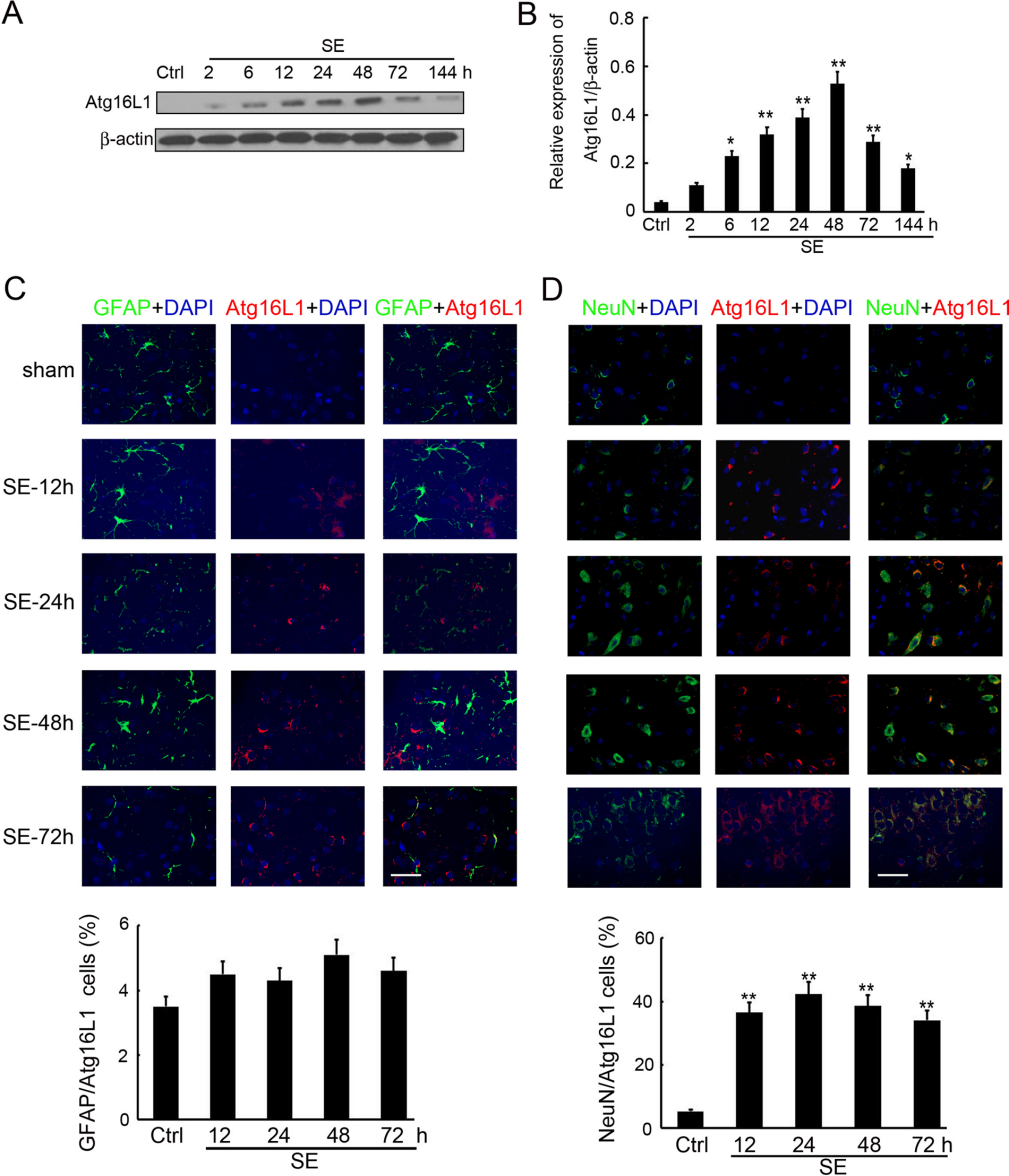
Expression and distribution of Atg16L1 after SE. (**A**) Western blotting analysis of Atg16L1 expression in the hippocampus of SE rats. Equal levels of protein samples (20 μg) were loaded, and β-actin served as a loading control. The relative optical density (Atg16L1/β-actin) were analyzed and represented as the mean ± SD (n = 3; **p < 0.01, compared with the ctrl group). (**B**) The cell type specificity of Atg16L1 was performed with double fluorescent immunolabeling (TRITC-labeled, red), accompanied with the astrocyte-specific marker GFAP (FITC-labeled, green). The nucleus was stained by DAPI (blue). Scale bar = 50 μm. The percent of GFAP and Atg16L1 positive cells was analyzed (n = 5; ns, no significant difference compared with the ctrl group). (**C**) The cell type specificity of Atg16L1 was performed with double fluorescent immunolabeling (TRITC-labeled, red), accompanied with the GFAP (FITC-labeled, green). The nucleus was stained by DAPI (blue). Scale bar = 50 μm. The percent of GFAP and Atg16L1 positive cells was analyzed (n = 5; **p < 0.01, compared with the ctrl group). (**D**) The cell type specificity of Atg16L1 was performed with double fluorescent immunolabeling (TRITC-labeled, red), accompanied with the neuron-specific marker NeuN (FITC-labeled, green). The nucleus was stained by DAPI (blue). Scale bar = 50 μm. The percent of NeuN and Atg16L1 positive cells was analyzed (n = 5; **p < 0.01, compared with the ctrl group).

Since Atg16L1 is another member of the Atg family with an important role during the formation of autophagosomes^32^, we also investigated its expression in the hippocampus of rats with SE, by Western blot analysis at 2, 6, 12, 24, 48, 72 and 144 h post insult. As shown in Fig. 3A, Atg16L1 expression was greatly increased from 6 to 144 h post insult, peaking at 48 h (Fig. 3A,B). To better analyse the expression and distribution of Atg16L1 in the hippocampus after SE insult, double immunofluorescence staining was performed to detect Atg16L1, as well as the astrocyte-specific marker GFAP or the neuronal-specific marker NeuN. The immunofluorescence staining supported the findings of Western blot analysis, showing an increase of Atg16L1 in the hippocampus from 12 to 72 h post SE (Fig. 3C,D). As was observed for Atg7, Atg16L1-immunoreactive cells mainly colocalized with neurons (Fig. 3D), while little Atg16L1 was found in astrocytes (Fig. 3C).

### Down-regulation of autophagy-related miR-96 in the hippocampus of SE rats

**Figure 4:**
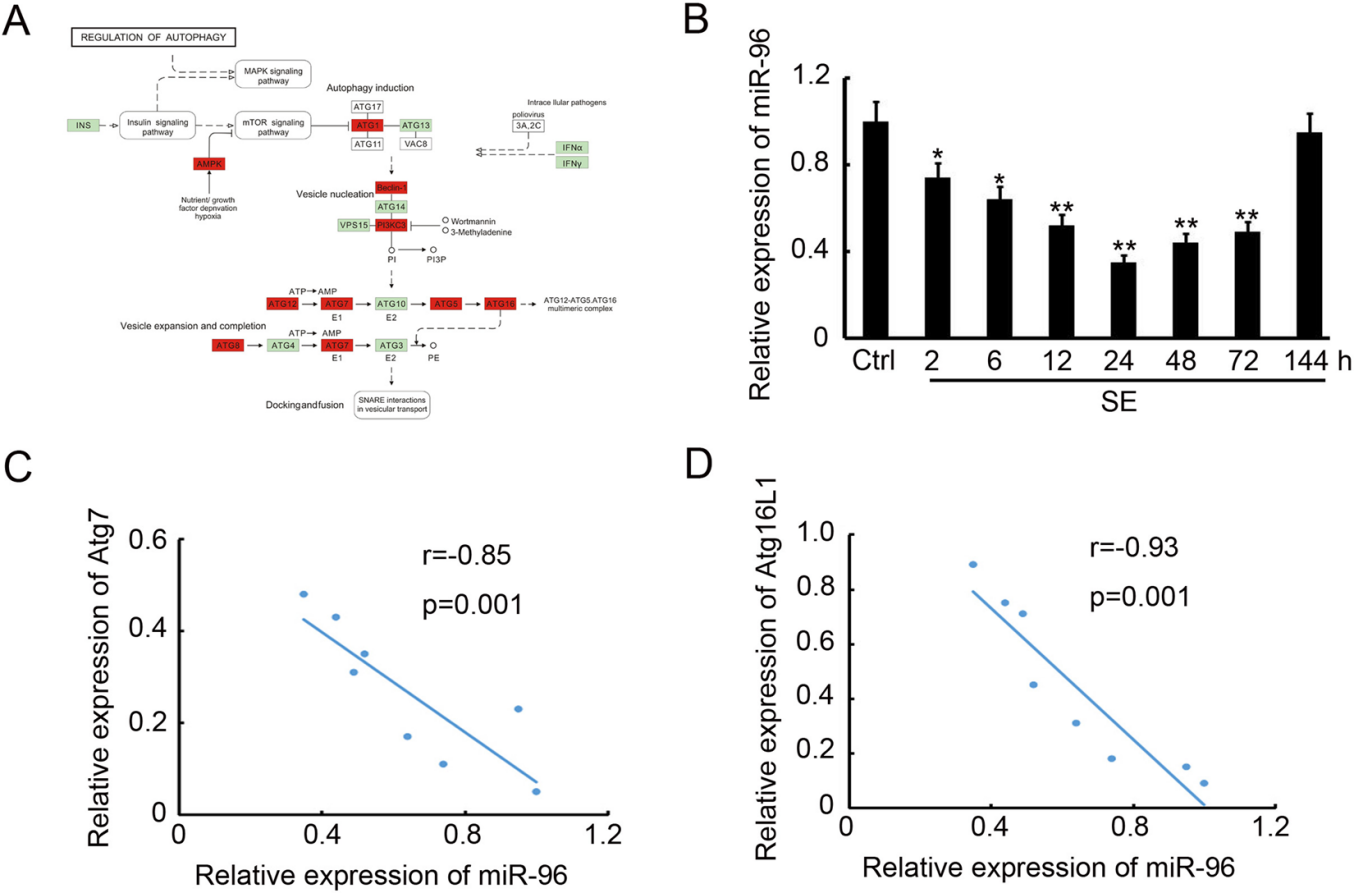
Downregulation of autophagy-related miR-96 in the hippocampus of SE rats. (**A**) The KEGG analysis of autophagy-related genes targeted by the down-regulated miRNAs. (**B**) The relative expression of miR-96 in the hippocampus at different time points after SE (n = 3; **p < 0.01, compared with the ctrl group). (**C**) The Pearson analysis of the correlation between miR-96 and Atg7 (r = −0.85, p = 0.001). (**D**) The Pearson analysis of the correlation between miR-96 and Atg16L1 (r = −0.93, p = 0.001).

In our previous study^23^, an miRNA array and related bioinformatics analysis by KEGG analysis^27–29^ indicated that miR-96 was one of the miRNAs with significantly altered expression and that the autophagy signalling pathway was regulated by these de-regulated miRNAs underlying epileptogenesis (Fig. 4A). We found that 9 out of 34 autophagy pathway genes were up-regulated by these down-regulated miRNAs, namely Atg7, Atg16L1, Atg5, Atg1, AMPK, Atg12, Atg8, Beclin-1 and PI3KC3 (Fig. 4A). A target prediction performed with the miRWalk database and other programs (miRanda, Sanger miRDB, RNAhybrid and Targetscan) predicted both Atg7 and Atg16L1 to be direct targets of miR-96. To further validate the expression of miR-96, total mRNA was isolated from the hippocampus of SE rats at 2, 6, 12, 24, 48, 72 and 144 h post insult and used for a quantitative reverse transcription polymerase chain reaction (qRT-PCR) assay. The results indicated that miR-96 levels decreased from 2 to 72 h after SE and returned to basal levels after 144 h (Fig. 4B). Pearson correlation analysis demonstrated that miR-96 expression was inversely correlated with Atg7 (r = −0.85, p = 0.001, Fig. 4C) and Atg16L1 (r = −0.93, p = 0.001, Fig. 4D) expression in the hippocampus of rats with SE.

### Overexpression of miR-96 attenuates SE-induced brain injury

**Figure 5:**
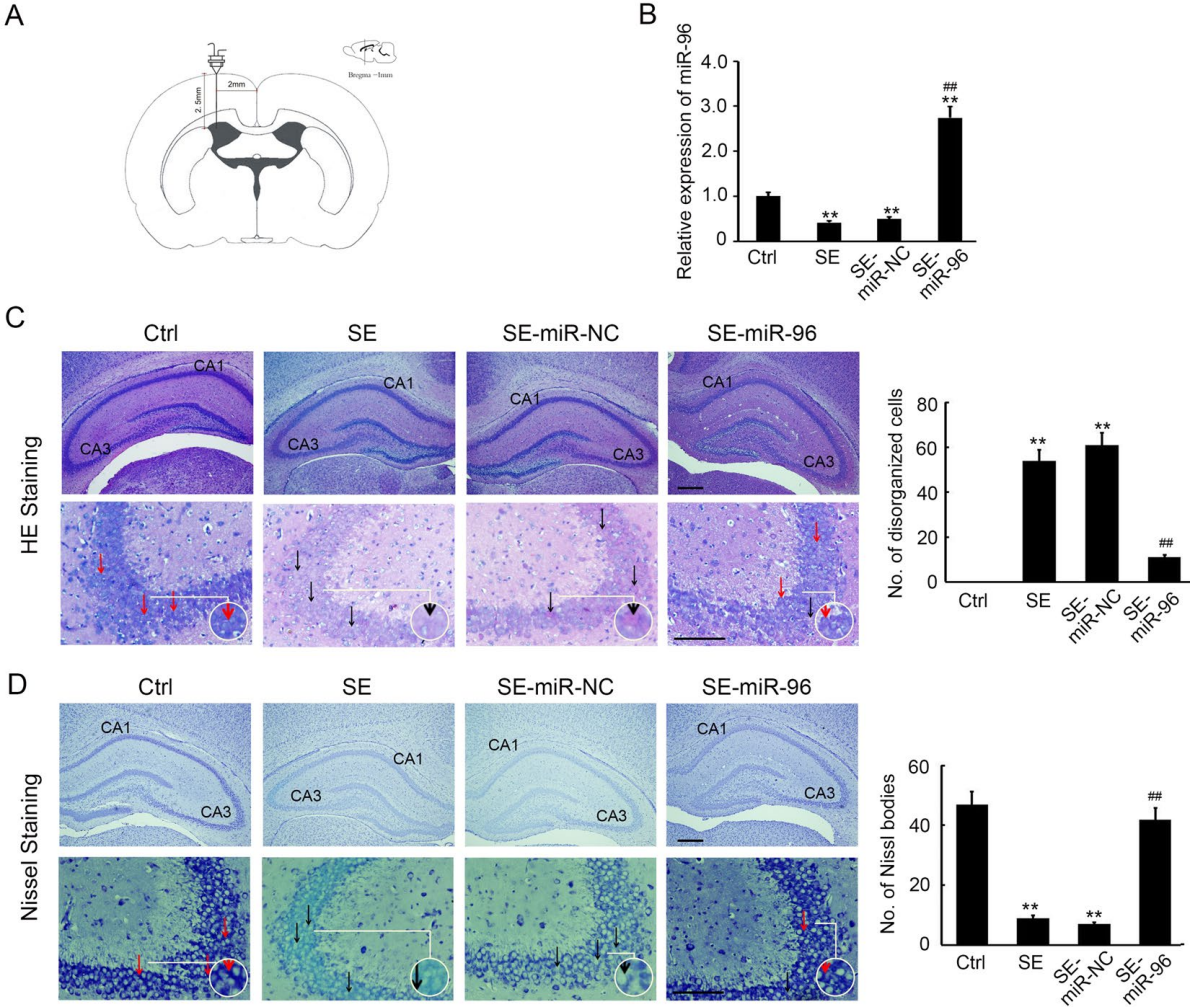
Overexpression of miR-96 attenuates SE-induced brain injury. (**A**) The model of miR-96 and miR-NC injection. (**B**) The relative expression of miR-96 in the hippocampus at different group (n = 5; **p < 0.01, compared with the ctrl group; ^##^p < 0.01, compared with the SE-miR-NC group). (**C**) H&E staining was performed to detect the histomorphology of the hippocampus. Scale bar = 100 μm. The number of disorganized cells in per frame were analyzed. The black arrow indicated the disorganized cells. The red arrow indicated the normal pyramidal cells. (**p < 0.01, compared with control group; ^##^p < 0.01, compared with the SE-miR-NC group) (**D**) Nissl staining was performed to detect the Nissl body in the hippocampus. Scale bar = 100 μm. The number of Nissl bodies in per frame were analyzed. The black arrow indicated the swelling, disorganized and membrane loss cells. The red arrow indicated the normal pyramidal cells. (**p < 0.01, compared with control group; ^##^p < 0.01, compared with the SE-miR-NC group).

To validate the potential protective role of miR-96 in SE-induced brain injury, miR-96 mimics were intracerebrally injected. As shown in Fig. 5A, 10 nmol miR-96 mimics or miR negative control (miR-NC), diluted in 5 μl phosphate buffer saline (PBS), were injected into the right cerebral hemisphere of SE rats using a 30-gauge needle with a 5 μl Hamilton syringe, 4 h prior to the SE insult. qRT-PCR results indicated a dramatic up-regulation of miR-96 expression in the miR-96 mimics injection group (Fig. 5B). Further, we investigated the morphological changes of the hippocampus with Hematoxylin and Eosin, and Nissl staining. As shown in Fig. 5C, HE staining indicated that the pyramidal cells in the CA1 and CA3 areas of SE and SE-miR-NC groups were swelling, disorganized and without nuclear and membrane structures. However, in the SE-miR-96 mimics injection group, the number of disorganized cells in the CA1 and CA3 areas was greatly reduced (Fig. 5C). Furthermore, Nissl staining was performed and revealed that only a small number of Nissl bodies was found in the SE and SE-miR-NC groups, accompanied with swelling, disorganized cells and membrane loss (Fig. 5D). These results indicate that injection of miR-96 mimics significantly prevents brain damage in SE rats.

### miR-96 inhibits expression of Atg7 and Atg16L1 in the hippocampus of SE rats

**Figure 6:**
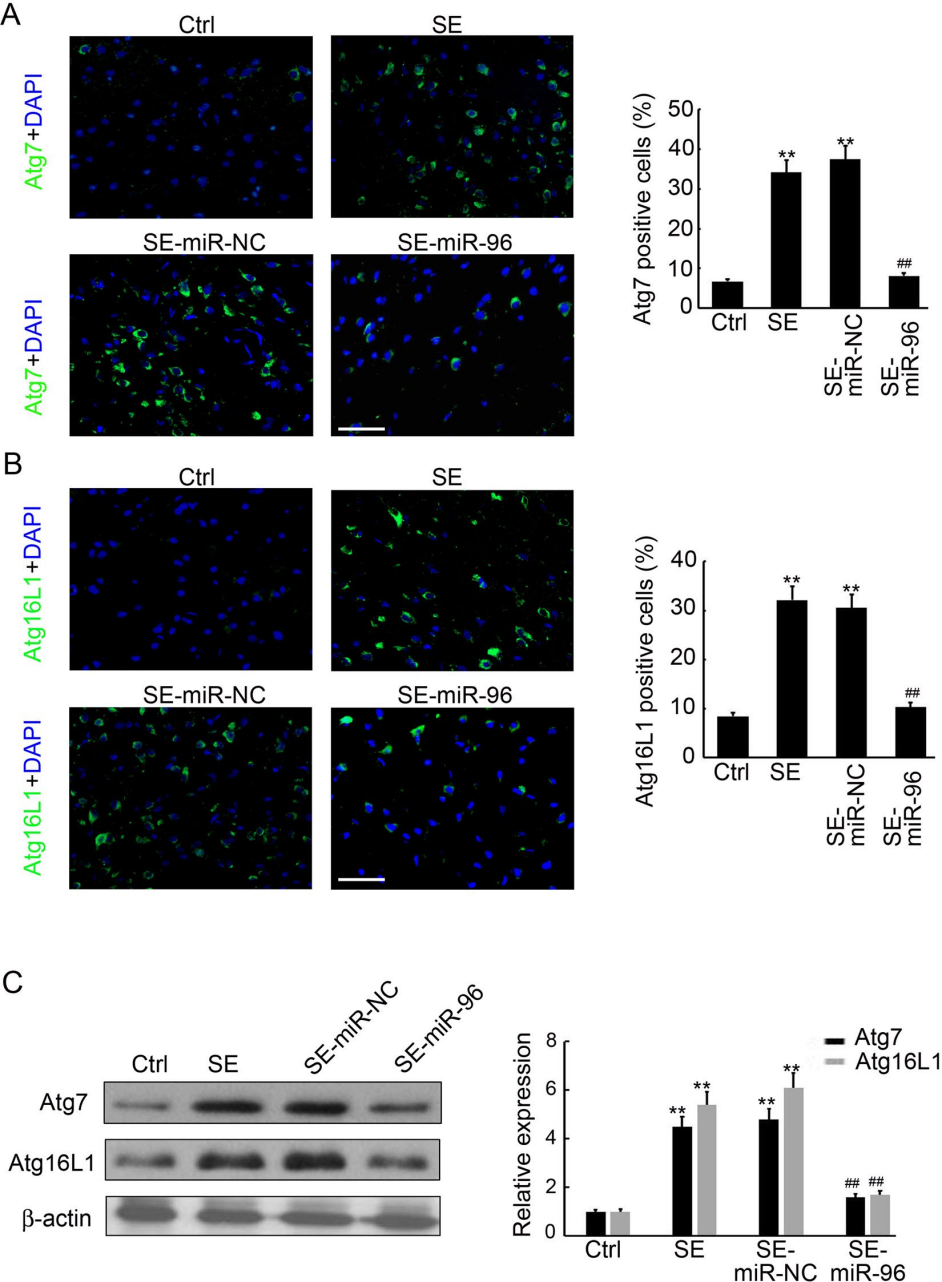
miR-96 inhibits hippocampus Atg7 and Atg16L1 expression in SE rats. (**A**) The cell type specificity of Atg7 was performed with fluorescent immunolabeling (FITC-labeled, green). The nucleus was stained by DAPI (blue). Scale bar = 50 μm. The percent of Atg7 positive cells was analyzed (n = 5; **p < 0.01, compared with the ctrl group; ^##^p < 0.01, compared with the SE-miR-NC group). (**B**) The cell type specificity of Atg16L1 was performed with fluorescent immunolabeling (FITC-labeled, green). The nucleus was stained by DAPI (blue). Scale bar = 50 μm. The percent of Atg16L1 positive cells was analyzed (n = 5; **p < 0.01, compared with the ctrl group; ^##^p < 0.01, compared with the SE-miR-NC group). (**C**) Western blotting analysis of Atg7 and Atg16L1 expression in the hippocampus of SE rats. Equal levels of protein samples (20 μg) were loaded, andβ-actin served as a loading control. The relative optical density were analyzed and represented as the mean ± SD (n = 3; **p < 0.01, compared with the ctrl group; ^##^p < 0.01, compared with the SE-miR-NC group).

To further elucidate the molecular mechanism underlying the protective role of miR-96 in SE-induced brain injury, we qualified the expression of miR-96 predicted targets Atg7 and Atg16L1 by immunofluorescence staining and Western blot analysis. As shown in Fig. 6A and B, overexpression of miR-96 significantly inhibited Atg7 and Atg16L1 expression in the hippocampus of SE rats. In the miR-96 injection group, only 8.5% and 10.4% cells were Atg7- and Atg16L1-immunoreactive, respectively (Fig. 6(A,B)). Western blot analysis also indicated that Atg7 and Atg16L1 protein levels were significantly reduced in the miR-96-injected hippocampus, when compared to the miR-NC-injected hippocampus (Fig. 6C). After normalisation to β-actin expression, we found a 59.4% decrease in Atg7 and a 66.7% decrease in Atg16L1 protein levels (Fig. 6C) in the miR-96-injected hippocampus. Taken together, these results show that injection of miR-96 significantly inhibited the expression of Atg7 and Atg16L1 in the hippocampus.

### miR-96 inhibits hippocampus autophagosome formation in SE rats

**Figure 7:**
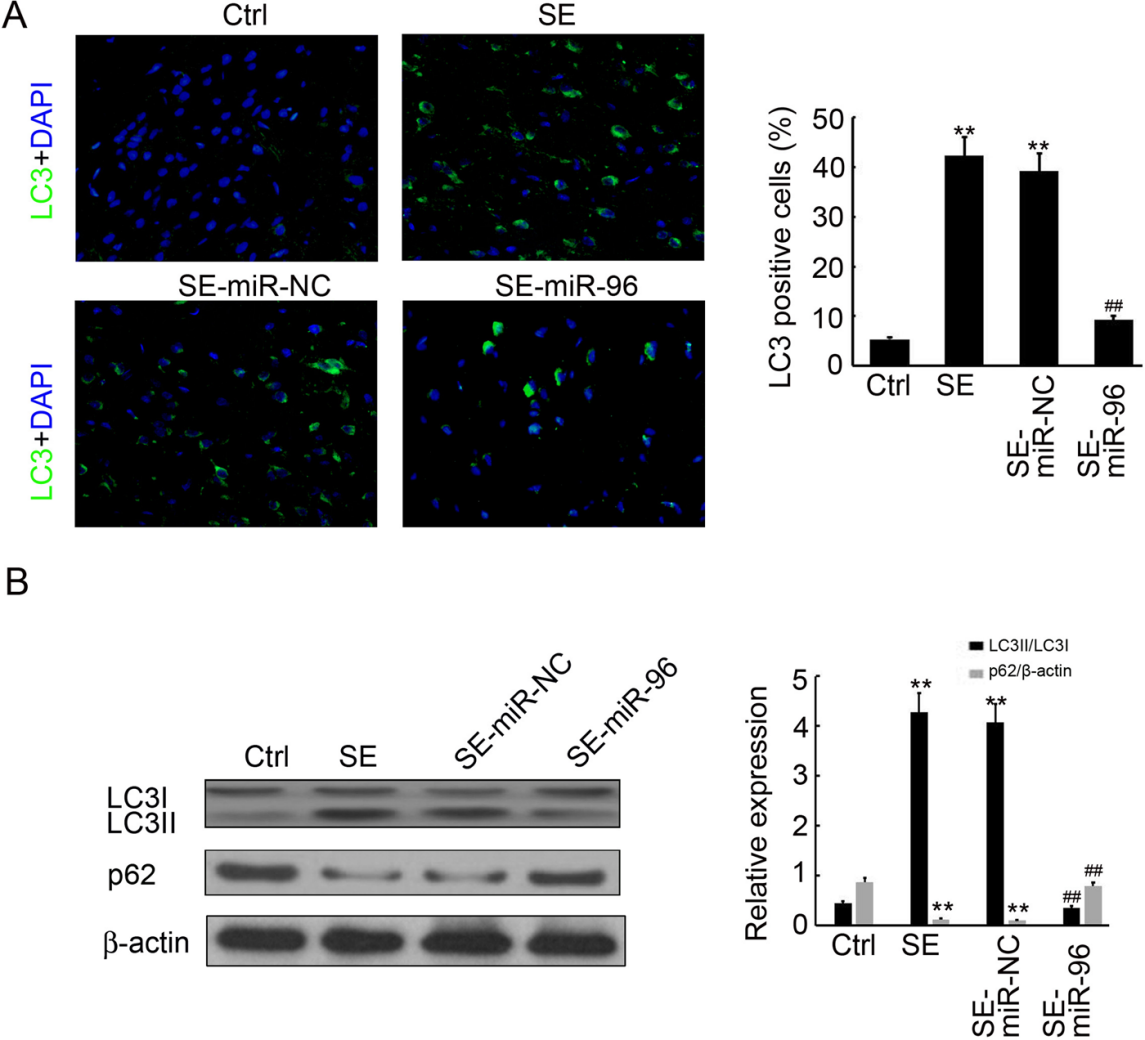
miR-96 inhibits hippocampus autophagosome formation in SE rats. (**A**) The cell type specificity of LC3 was performed with fluorescent immunolabeling (FITC-labeled, green). The nucleus was stained by DAPI (blue). Scale bar = 50 μm. The percent of Atg7 positive cells was analyzed (n = 5; **p < 0.01, compared with the ctrl group; ^##^p < 0.01, compared with the SE-miR-NC group). (**B**) Western blotting analysis of LC3 and p62 expression in the hippocampus of SE rats. Equal levels of protein samples (20 μg) were loaded, and β-actin served as a loading control. The relative optical density were analyzed and represented as the mean ± SD (n = 3; **p < 0.01, compared with the ctrl group; ^##^p < 0.01, compared with the SE-miR-NC group).

The clear down-regulation of Atg7 and Atg16L1 expression in the miR-96-injected hippocampus motivated us to investigate changes in autophagosome formation in the groups submitted to the different treatments. Immunofluorescence staining indicated that less LC3-positive cells were found in the miR-96-injected hippocampus, a 73.4% decrease (Fig. 7A). Western blot analysis also showed a 64.4% reduction in the ratio of LCII/LC3I and a 3.8 fold upregulation of p62 expression in the miR-96 mimics-injected hippocampus, when compared to the negative control (Fig. 7B). In conclusion, injection of miR-96 could inhibit autophagosome formation in the hippocampus.

### Rapamycin negated miR-96 mediated brain injury attenuation

**Figure 8:**
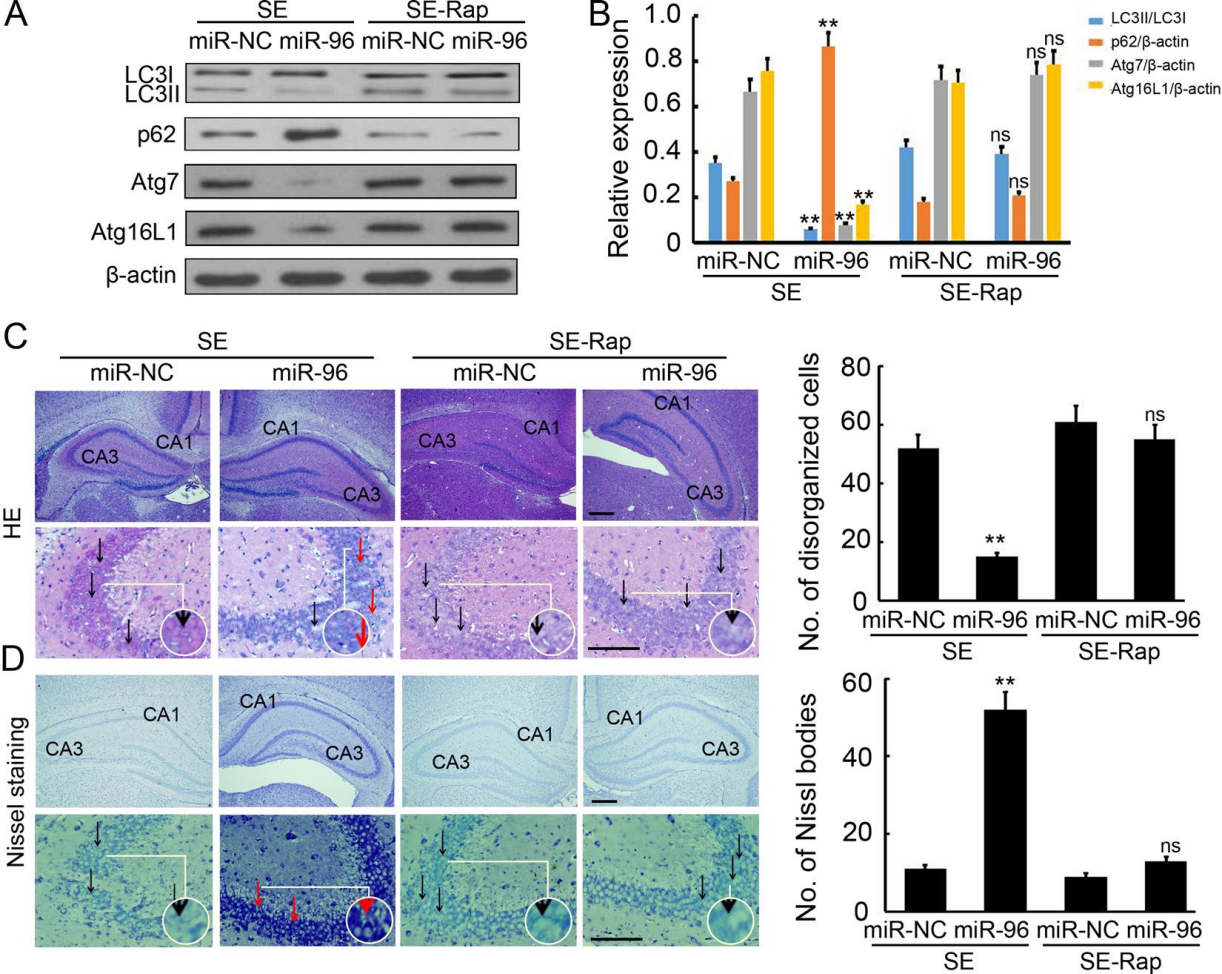
Rapamycin negated miR-96 mediated brain injury attenuation. (**A**) Western blotting analysis of LC3, p62, Atg7 and Atg16L1 expression in the hippocampus of SE rats. Equal levels of protein samples (20 μg) were loaded, and β-actin served as a loading control. (**B**) The relative optical density were analyzed and represented as the mean ± SD (n = 3; **p < 0.01, compared with the SE-miR-NC group; ns, no significant difference, compared with the SE-Rap-miR-NC group). (**C**) H&E staining was performed to detect the histomorphology of the hippocampus. Scale bar = 100 μm. The number of disorganized cells in per frame were analyzed. The black arrow indicated the disorganized cells. The red arrow indicated the normal pyramidal cells. (**p < 0.01, compared with the SE-miR-NC group; ns, no significant difference, compared with the SE-Rap-miR-NC group). (**D**) Nissl staining was performed to detect the Nissl body in the hippocampus. Scale bar = 100 μm. The number of Nissl bodies in per frame were analyzed. The black arrow indicated the swelling, disorganized and membrane loss cells. The red arrow indicated the normal pyramidal cells. (**p < 0.01, compared with the SE-miR-NC group; ns, no significant difference, compared with the SE-Rap-miR-NC group).

To further elucidate the autophagy effects underlying the protective role of miR-96 in SE-induced brain injury, we employed Rapamycin to treat miR-NC or miR-96-mimcs injected SE rats. As shown in Fig. 8A,B, Rapamycin attenuated miR-96 mediated reduction in the ratio of LC3II/LC3I and upregulation of p62 expression (Fig. 8A,B). It’s indicated that autophagy in the CNS of SE rats was induced by Rapamycin. Furthermore, HE staining and Nissl staining demonstrated that there were no significant difference on disorganized cells and Nissl bodies between SE-Rapamycin-miR-NC injection group and SE-Rapamycin-miR-96 mimics injection group (Fig. 8C,D). Whereas more disorganized cells and less Nissl bodies were found in the SE-miR-96 mimics injection group, compared with SE-miR-NC injection group (Fig. 8C,D). In conclusion, Rapamycin negated miR-96 mediated brain injury attenuation through inducing autophagosome formation.

## Discussion

SE is the most severe and lethal form of epilepsy^5–7^; hence, special attention should be paid to developing novel targets for its therapy and diagnosis. In the present study, we investigated the potential of autophagy-related miRNAs as diagnosis and treatment target markers for SE in immature rats. We showed that autophagosome were found in neurons after SE, together with increasing expression of Atg7 and Atg16L1. Furthermore, our results indicated that a reduction of miR-96 was inversely correlated with the up-regulation of Atg7 and Atg16L1 expression. Overexpression of miR-96 through intracerebral injection had a preventive role on SE-induced brain damage, by inhibiting Atg7 and Atg16L1 expression, followed by autophagosome formation in immature rats. Our study suggests that miR-96 gets its protective role during the development of SE, thus providing a novel therapy target for SE therapy.

Autophagy is like a ‘double-edged sword’^17, 33^, as it probably facilitates both the promotion and prevention of epilepsy. While inactivation of Atg7 is sufficient to induce epilepsy due to impaired autophagy^34^, CBI, vitamin E and ascorbic acid may potentially prevent seizure-induced neurobehavioral deficits by inhibiting autophagy^35, 36^. We found that SE increased the expression of LC3, Atg7 and Atg16L1, in a time-dependent manner, in immature rats. Previous studies reported the regulatory role of autophagy in neurodegeneration and astroglial death in epilepsy^16, 37^, but to our knowledge, did not investigate the distribution of autophagosome in the hippocampus of immature rats with SE. In the present study, double immunofluorescence was performed and indicated that autophagosome localised in the neurons (Fig. 1), but not the astrocytes, associated with the induction of Atg7 (Fig. 2) and Atg16L1 (Fig. 3) expression in the hippocampus of immature rats with SE. These results lay the foundation for a better understanding of the association between autophagy and SE and the mechanism underlying this association.

Recently, various studies were performed to elucidate the function of miRNAs in CNS disease by global profiling techniques. These identified more than 100 differentially expressed miRNAs associated with epileptogenesis, which affect neurogenesis, inflammation and apoptosis of the CNS^38, 39^. One paper reported 19 up-regulated and 7 down-regulated miRNAs in the adult rat hippocampus following lithium–pilocarpine-induced SE, using a rat miRNA array and differential analysis^40^; however, few recent studies have characterized the miRNA expression patterns in developing hippocampi following SE. Our previous study have examined miRNA expression profiles in the hippocampi of immature rats following SE and reported that 30 up-regulated miRNAs and 21 down-regulated miRNAs have differential expression, when compared to a control group. Furthermore, Kyoto Encyclopaedia of Genes and Genomes (KEGG) analysis was performed to assess the predicted target genes of the down-regulated miRNAs and the results suggested that autophagy was involved in the cellular processes of epileptogenesis, which are regulated by the down-regulated miRNAs. The target prediction indicated that Atg7 and Atg16L1 were both direct targets of miR-96. Previous studies indicated that up-regulation of miR-146a was detected in rat models and patients with temporal lobe epilepsy (TLE)^14, 41, 42^, playing a role in the modification of posttranscriptional inflammation. Additionally, Hu and co-workers showed that the up-regulation of miR-34a in the rat hippocampus activated the caspase-3 protein and that antagomirs (synthetic miRNA inhibitors) of miRNA-34a could block miR-34a–Bcl-2–caspase-3 signalling, resulting in a neuro-protective effect^43^. Therefore, to our knowledge, our study is the first to show miRNA (miR-96) to be involved in autophagy during epileptogenesis. Pearson correlation analysis revealed that miR-96 expression was inversely correlated with Atg7 (r = −0.85, p = 0.001) and Atg16L1 (r = -0.93, p = 0.001) expression in the hippocampus of rats with SE (Fig. 4E). Furthermore, injection of miR-96 mimics significantly prevented SE rats from brain damage (Fig. 5) by inhibiting Atg7 and Atg16L1 expression (Fig. 6) and autophagosome formation (Fig. 7) in the hippocampus. These results indicate the possibility of novel biomarkers and therapeutic strategies for pediatric SE.

In conclusion, our study is the first to demonstrate an increase in autophagosomes in neurons after SE, accompanied by the induction of Atg7 and Atg16L1 expression, which result from a reduction in miR-96 levels. Overexpression of miR-96 significantly prevents SE rats from brain damage by inhibiting the Atg7 and Atg16L1 expression and consequently, autophagosome formation in the hippocampus. This suggests that miR-96 might be a potential target for therapy of pediatric SE.

## Electronic supplementary material

Supplementary information

## References

[1] Chang BS, Lowenstein DH (2003). Epilepsy. The New England journal of medicine.

[2] McNamara JO, Huang YZ, Leonard AS (2006). Molecular signaling mechanisms underlying epileptogenesis. Science Signaling.

[3] Pitkänen A, Lukasiuk K (2011). Mechanisms of epileptogenesis and potential treatment targets. The Lancet Neurology.

[4] Rojas A, Ganesh T, Lelutiu N, Gueorguieva P, Dingledine R (2015). Inhibition of the prostaglandin EP2 receptor is neuroprotective and accelerates functional recovery in a rat model of organophosphorus induced status epilepticus. Neuropharmacology.

[5] Di Bonaventura C (2008). Status epilepticus in epileptic patients. Related syndromes, precipitating factors, treatment and outcome in a video-EEG population-based study. Seizure.

[6] Jallon P (2004). Mortality in patients with epilepsy. Current opinion in neurology.

[7] Logroscino G (2005). Mortality after a first episode of status epilepticus in the United States and Europe. Epilepsia.

[8] Levine B, Klionsky DJ (2004). Development by self-digestion: molecular mechanisms and biological functions of autophagy. Developmental cell.

[9] Klionsky DJ (2007). Autophagy: from phenomenology to molecular understanding in less than a decade. Nature reviews. Molecular cell biology.

[10] Palumbo S, Miracco C, Pirtoli L, Comincini S (2014). Emerging roles of microRNA in modulating cell-death processes in malignant glioma. Journal of cellular physiology.

[11] Zhu H (2009). Regulation of autophagy by a beclin 1-targeted microRNA, miR-30a, in cancer cells. Autophagy.

[12] Ucar A (2012). The miRNA-212/132 family regulates both cardiac hypertrophy and cardiomyocyte autophagy. Nature communications.

[13] Massey DC, Parkes M (2007). Genome-wide association scanning highlights two autophagy genes, ATG16L1 and IRGM, as being significantly associated with Crohn’s disease. Autophagy.

[14] Alvarez-Erviti L (2013). Influence of microRNA deregulation on chaperone-mediated autophagy and alpha-synuclein pathology in Parkinson’s disease. Cell death & disease.

[15] Criado O (2012). Lafora bodies and neurological defects in malin-deficient mice correlate with impaired autophagy. Human molecular genetics.

[16] Ni H, Feng X, Gong Y, Tao LY, Wu XR (2011). Acute phase expression pattern of ZnTs, LC3, and beclin-1 in rat Hippocampus and its regulation by 3-methyladenine following recurrent neonatal seizures. Biological trace element research.

[17] Gan J, Qu Y, Li J, Zhao F, Mu D (2015). An evaluation of the links between microRNA, autophagy, and epilepsy. Reviews in the neurosciences.

[18] Ni H, Yan JZ, Zhang LL, Feng X, Wu XR (2012). Long-term effects of recurrent neonatal seizures on neurobehavioral function and related gene expression and its intervention by inhibitor of cathepsin B. Neurochemical research.

[19] Baek D (2008). The impact of microRNAs on protein output. Nature.

[20] Lim LP (2005). Microarray analysis shows that some microRNAs downregulate large numbers of target mRNAs. Nature.

[21] Jackson AL, Levin AA (2012). Developing microRNA therapeutics: approaching the unique complexities. Nucleic acid therapeutics.

[22] Henshall DC (2014). MicroRNA and epilepsy: profiling, functions and potential clinical applications. Current opinion in neurology.

[23] Ahsan H, Thomas DC (2004). Lung cancer etiology: independent and joint effects of genetics, tobacco, and arsenic. Jama.

[24] Drion CM (2016). Effects of rapamycin and curcumin treatment on the development of epilepsy after electrically induced status epilepticus in rats. Epilepsia.

[25] Tizon B (2010). Induction of autophagy by cystatin C: a mechanism that protects murine primary cortical neurons and neuronal cell lines. PloS one.

[26] Dai L (2016). SARI inhibits angiogenesis and tumour growth of human colon cancer through directly targeting ceruloplasmin. Nature communications.

[27] Kanehisa M, Furumichi M, Tanabe M, Sato Y, Morishima K (2017). KEGG: new perspectives on genomes, pathways, diseases and drugs. Nucleic acids research.

[28] Kanehisa M, Sato Y, Kawashima M, Furumichi M, Tanabe M (2016). KEGG as a reference resource for gene and protein annotation. Nucleic acids research.

[29] Kanehisa M, Goto S (2000). KEGG: kyoto encyclopedia of genes and genomes. Nucleic acids research.

[30] Luo J (2014). Autophagy and ethanol neurotoxicity. Autophagy.

[31] Ra EA (2016). TRIM31 promotes Atg5/Atg7-independent autophagy in intestinal cells. Nature communications.

[32] Liang J (2015). Myristoylation confers noncanonical AMPK functions in autophagy selectivity and mitochondrial surveillance. Nature communications.

[33] Shintani T, Klionsky DJ (2004). Autophagy in health and disease: a double-edged sword. Science (New York, N.Y.).

[34] McMahon J (2012). Impaired autophagy in neurons after disinhibition of mammalian target of rapamycin and its contribution to epileptogenesis. The Journal of neuroscience: the official journal of the Society for Neuroscience.

[35] Cao L (2009). Vitamin E inhibits activated chaperone-mediated autophagy in rats with status epilepticus. Neuroscience.

[36] Dong Y (2013). Ascorbic acid ameliorates seizures and brain damage in rats through inhibiting autophagy. Brain research.

[37] Ryu HJ, Kim JE, Yeo SI, Kang TC (2011). p65/RelA-Ser529 NF-kappaB subunit phosphorylation induces autophagic astroglial death (Clasmatodendrosis) following status epilepticus. Cellular and molecular neurobiology.

[38] Dogini DB, Avansini SH, Vieira AS, Lopes-Cendes I (2013). MicroRNA regulation and dysregulation in epilepsy. Frontiers in cellular neuroscience.

[39] Wang J (2015). Genome-wide circulating microRNA expression profiling indicates biomarkers for epilepsy. Scientific reports.

[40] Hu K (2012). MicroRNA expression profile of the hippocampus in a rat model of temporal lobe epilepsy and miR-34a-targeted neuroprotection against hippocampal neurone cell apoptosis post-status epilepticus. BMC Neurosci.

[41] Iyer A (2012). MicroRNA-146a: a key regulator of astrocyte-mediated inflammatory response. PloS one.

[42] Omran A (2012). Interleukin-1beta and microRNA-146a in an immature rat model and children with mesial temporal lobe epilepsy. Epilepsia.

[43] Hu K (2012). MicroRNA expression profile of the hippocampus in a rat model of temporal lobe epilepsy and miR-34a-targeted neuroprotection against hippocampal neurone cell apoptosis post-status epilepticus. BMC neuroscience.

