# Supplementary material for: miR-96 attenuates status epilepticus-induced brain injury by directly targeting Atg7 and Atg16L1
Source: Sci Rep. 2017 Aug 31;7:10270. doi: 10.1038/s41598-017-10619-0 (PMC5579030; doi:10.1038/s41598-017-10619-0)

miR-96 attenuates status epilepticus-induced brain injury by directly targeting Atg7 and Atg16L1

Jing Gan[1,2], Qianyun Cai[1,2], Yi Qu [1,2], Fengyan Zhao[1,2], Rong Luo[1,2], Chaomin Wan[1,2], Dezhi Mu[1,2,3*]

[1]Department of Pediatrics, West China Second University Hospital, Sichuan University, Chengdu 610041, China

[2]Key Laboratory of Obstetric & Gynecologic and Pediatric Diseases and Birth Defects of Ministry of Education, Sichuan University, Chengdu 610041, China

[3]Department of Pediatrics, University of California, San Francisco, CA94143, USA

Corresponding author: Dezhi Mu MD, PhD

Department of Pediatrics, West China Second University Hospital, Sichuan University

Chengdu, Sichuan 610041, P. R. China

Email: mudz@scu.edu.cn

Tel: +86-28-85559065

Figure 1

The sample order: Ctrl, SE-2, 6, 12, 24, 48, 72, 144h

LC3

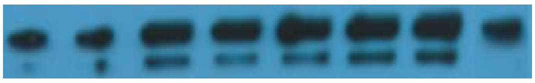

Beclin-1

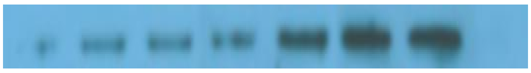

P62

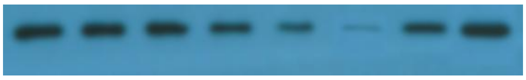

β-actin

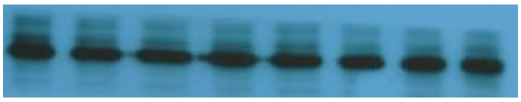

Figure 2

The sample order: Ctrl, SE-2, 6, 12, 24, 48, 72, 144h

Atg7

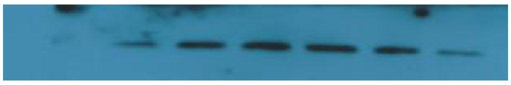

β-actin

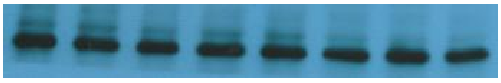

Figure 3

The sample order: Ctrl, SE-2, 6, 12, 24, 48, 72, 144h

Atg16 L1

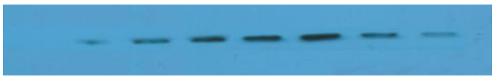

β-actin

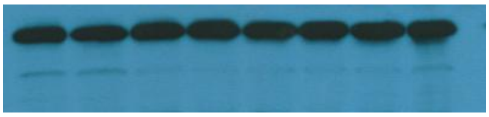

Figure 6

The sample order: Ctrl, SE, SE-miR-NC, SE-miR-96

Atg7

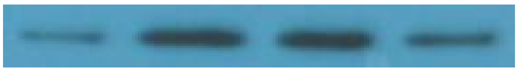

Atg16L1

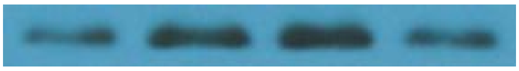

β-actin

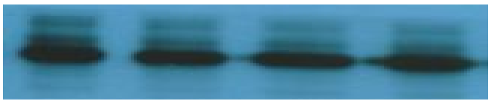

Figure 7

The sample order: Ctrl, SE, SE-miR-NC, SE-miR-96

LC3

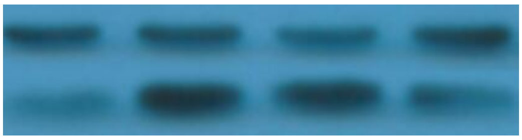

P62

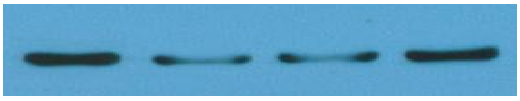

β-actin

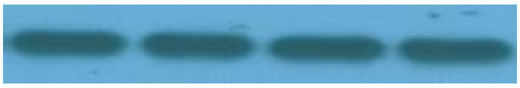

Figure 8

The sample order: SE-miR-NC, SE-miR-96, SE-Rap-miR-NC, SE-Rap-miR-96

LC3

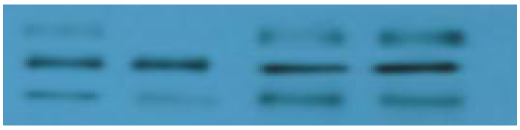

P62

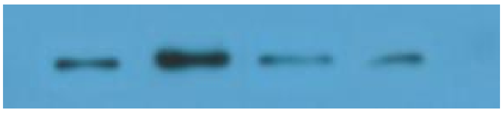

β-actin

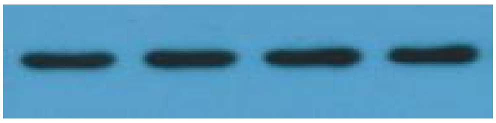

Supplement: Supplementary file 1 — Supplementary information [file 41598_2017_10619_MOESM1_ESM.pdf]
